# Supplementary material for: Effectiveness and Safety of Ayurvedic Medicines in Type 2 Diabetes Mellitus Management: A Systematic Review and Meta-Analysis
Source: Front Pharmacol. 2022 Jun 8;13:821810. doi: 10.3389/fphar.2022.821810 (PMC9213670; doi:10.3389/fphar.2022.821810)
Supplement: Supplementary file 1 [file DataSheet1.zip › Supplementary Material/Forest and Funnel Plots/Punica granatum/BMI.pdf]

Punica granatum - BMI

Study	Intervention N	Intervention Mean	Intervention SD	Comparator N	Comparator Mean	Comparator SD	Mean Diff. with 95% CI	Weight (%)
Babaeian 2013	23	28.22	2.75	22	28.58	3.05	-0.36 [-2.06, 1.34]	27.73
Sohrab 2014, Sohrab 2015	22	29.40	3.90	22	28.40	4.40	1.00 [-1.46, 3.46]	13.35
Faghihimani 2017	37	27.00	2.30	37	26.00	3.10	1.00 [-0.24, 2.24]	50.59
Grabež 2020	19	31.94	4.90	18	32.58	4.76	-0.64 [-3.76, 2.48]	8.33
**Overall**							0.49 [-0.42, 1.39]	

Heterogeneity: $\tau^2 = 0.02$, $I^2 = 1.64\%$, $H^2 = 1.02$

Test of $\theta_i = \theta_j$: $Q(3) = 2.28$, $p = 0.52$

Test of $\theta = 0$: $z = 1.06$, $p = 0.29$

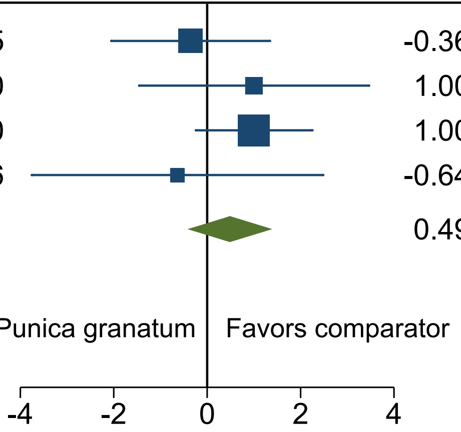

Random-effects REML model
